# Supplementary material for: Survival Outcomes Associated with the Location of BRCA Mutations in Ovarian Cancer: A Systematic Review and Meta-Analysis
Source: Cancers (Basel). 2025 May 14;17(10):1661. doi: 10.3390/cancers17101661 (PMC12109784; doi:10.3390/cancers17101661)
Supplement: Supplementary file 1 [file cancers-17-01661-s001.zip › Supplement S1_Search Strategy.pdf]

Search strategy

Search strategy for MEDLINE

#1. “ovarian” [All fields]

#2. “ovary” [All fields]

#3. (#1) OR #2

#4. “cancer” [All fields]

#5. “carcinoma” [All fields]

#6. “neoplasm” [All fields]

#7. “malignancy” [All fields]

#8. “tumor” [All fields]

#9. ((((#4) OR #5) OR #6) OR #7) OR #8

#10. “survival”

#11. “prognosis”

#12. “outcome”

#13. “mortality”

#14. “recurrence”

#15. ((((#10) OR #11) OR #12) OR #12) OR #13) OR #14

#16. “BRCA”

#17. “BRCA1”

#18. “BRCA2”

#19. “BRCA1/2”

#20. ((((#16) OR #17) OR #18) OR #19)

#21. ((((#3) AND #9) AND #15) AND #20

Search strategy for EMBASE

#1. ‘ovarian’/exp

#2. ‘ovary’/exp

#3. #1 OR #2

#4. ‘cancer’/exp

#5. ‘carcinoma’/exp

#6. ‘neoplasm’/exp

#7. ‘malignancy’/exp

#8. ‘tumor’/exp

#9. #4 OR #5 OR #6 OR #7 OR #8

#10. ‘survival’/exp

#11. ‘prognosis’/exp

#12. ‘outcome’/exp

#13. ‘mortality’/exp

#14. ‘recurrence’/exp

#15. #10 OR #11 OR #12 OR #13 OR #14

#16. ‘BRCA’/exp

#17. ‘BRCA1’/exp

#18. ‘BRCA2’/exp

#19. ‘BRCA1/2’/exp

#20. #16 OR #17 OR #18 OR #19

#21. #3 AND #9 AND #15 AND #20

Search strategy for the Cochrane Library#1. “ovarian”

#2. “ovary”

#3. #1 or #2

#4. “cancer”

#5. “carcinoma”

#6. “neoplasm”

#7. “malignancy”

#8. “tumor”

#9. #4 or #5 or #6 or #7 or #8

#10. “survival”

#11. “prognosis”

#12. “outcome”

#13. “mortality”

#14. “recurrence”

#15. #10 or #11 or #12 or #13 or #14

#16. “BRCA”

#17. “BRCA1”

#18. “BRCA2”

#19. “BRCA1/2”

#20. #16 OR #17 OR #18 OR #19

#21. #3 AND #9 AND #15 AND #20

Date of Search: August 13, 2024

Result: 6,712 articles found
