# Supplementary figures and images for: Survival Outcomes Associated with the Location of BRCA Mutations in Ovarian Cancer: A Systematic Review and Meta-Analysis
Source: Cancers (Basel). 2025 May 14;17(10):1661. doi: 10.3390/cancers17101661 (PMC12109784; doi:10.3390/cancers17101661)

Figure S1. Funnel plot for PFS

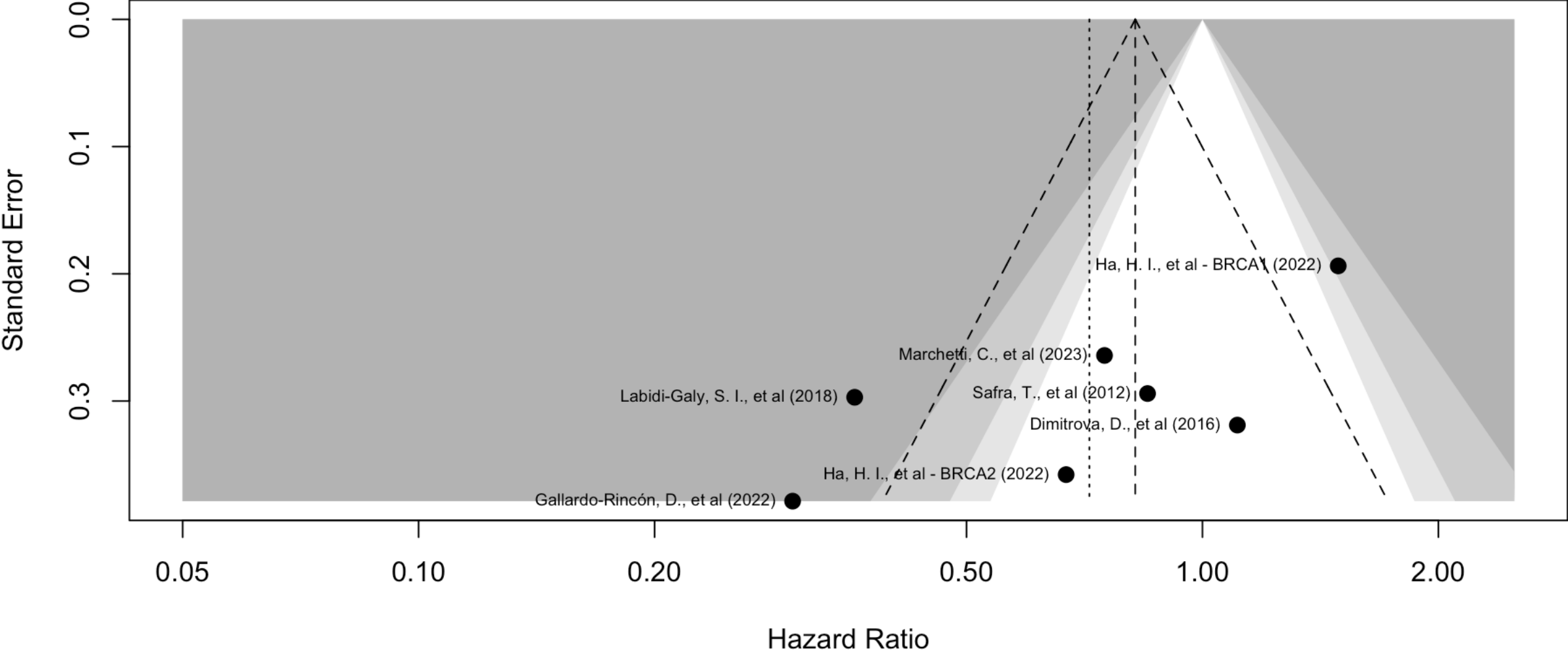

Figure S2. Funnel plot for OS

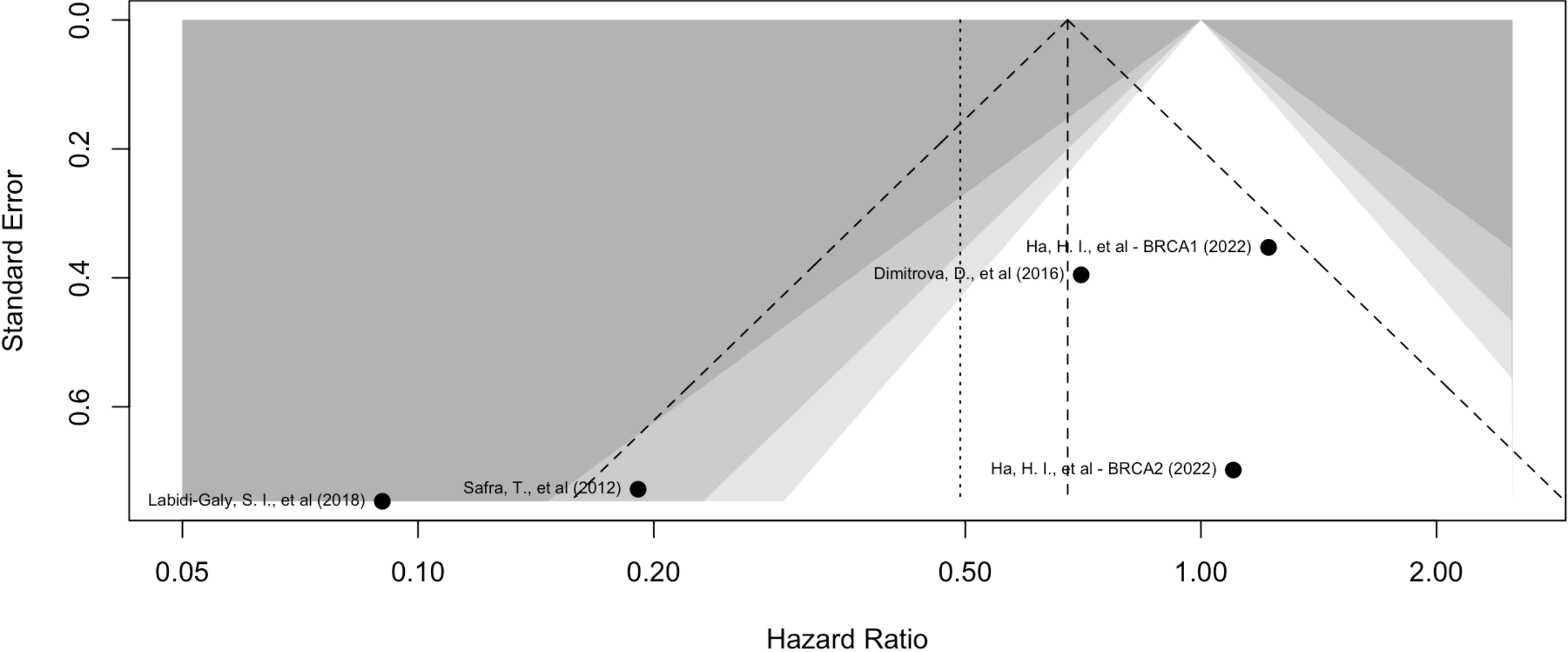

Supplement: Supplementary file 1 [file cancers-17-01661-s001.zip › Supplementary Figure S1 and S2.pdf]

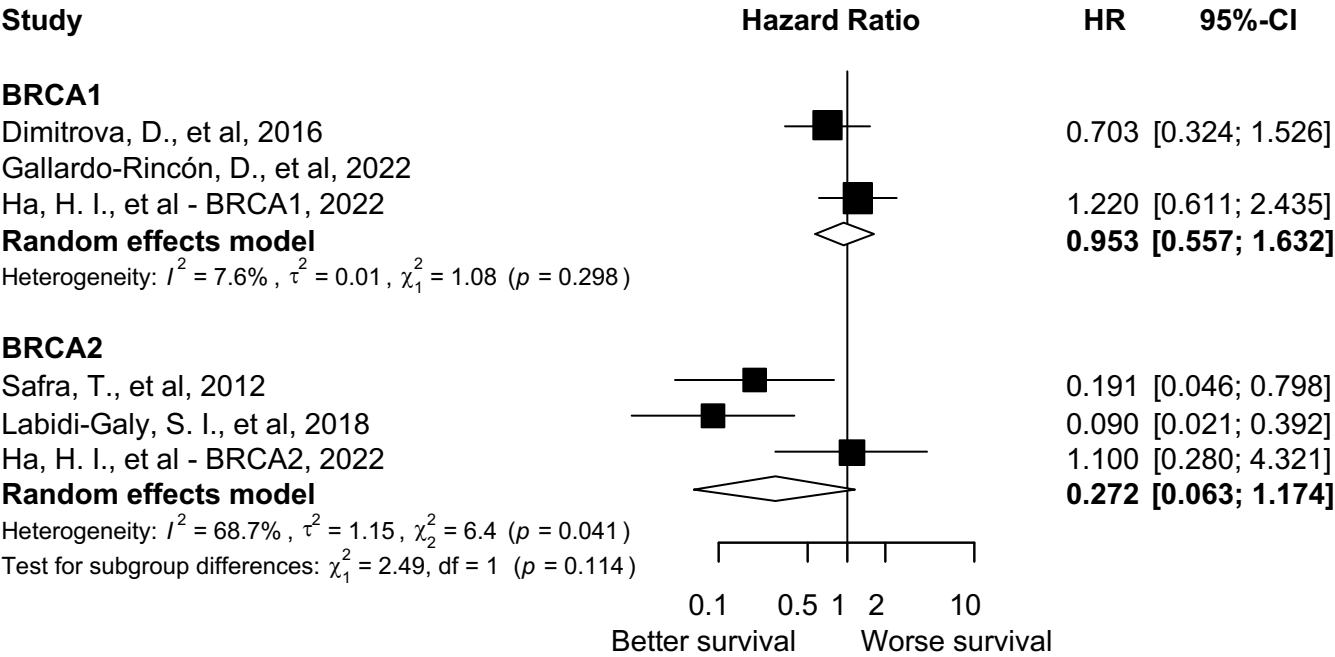

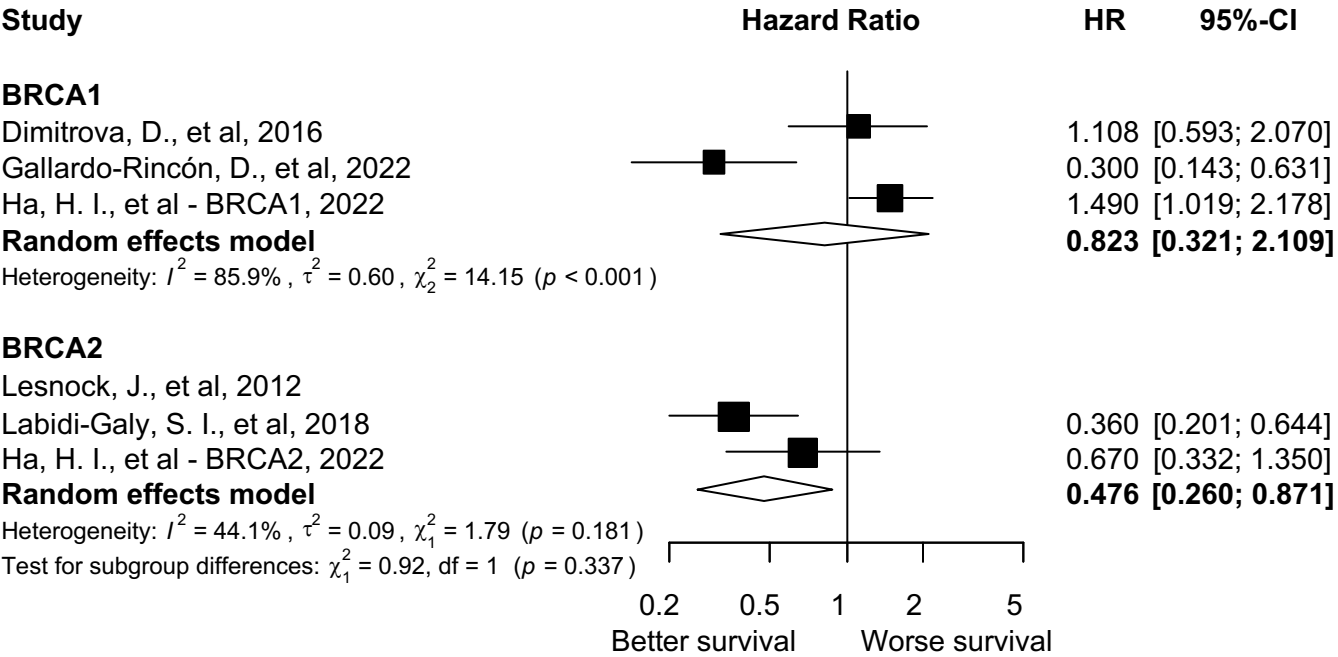

Supplement: Supplementary file 1 [file cancers-17-01661-s001.zip › Supplementary Figure S3.pdf]
